# Annotations

**Published:** 1910-12-24

**Authors:** 


### ANNOTATIONS.

### The late Professor Mosso.

The death is announced from Turin of Professor Angelo Mosso, the eminent physiologist, at the age of sixty-four. Born in 1846, he was educated in the University of Turin, his native town, taking his degree of Doctor of Medicine in 1866. Later he studied at Florence, in Germany, and at Paris. He was appointed Professor at the Faculty of Medicine of Turin in 1876, and four years later was called to the Chair of Physiology at Rome, which he held until a short time ago, when he found himself obliged to resign it owing to a painful and incurable disease. In 1904 he was made a Senator of the Kingdom of Italy. He devoted much of his time to the study of physiological problems, and made himself a European reputation by his researches into the temperature of the human brain, the physical phenomena of the emotions, and the effects of high altitudes upon the human organism, with special reference to the causation of mountain sickness. In this last connection he startled the scientific world somewhat by his theory that the principal cause of respiratory troubles in high altitudes was the relative rarefaction of the air in carbonic acid and not in oxygen, as usually believed. This theory has of late years gained many adherents. He was the inventor of several scientific instruments, including the plethysmograph and ergograph, which bear his name, and the author of several popular works on hygiene, in addition to his many contributions to medical periodicals, and his physiological books. He filled in his time after quitting his Chair by directing the works of excavation being carried out at Gnossus in Crete, his last literary labours being devoted to a description of the ancient civilisation unearthed by the workers under his command.

### Health Resorts and Gambling.

Dr. Jules Felix publishes, in *La Gazette Médicale Belge*, an article on the modern watering-place, in which he attacks the lethargy of his own countrymen in the matter of scientific and rational exploitation of the Belgian watering-places. An idea seems to have got about in Belgium that it is impossible to make a health-resort attractive, that is to say a paying concern, unless some facilities for gambling are provided. This idea is hopelessly wrong. Statistics collected by Bismarck in 1873, when attempting to suppress gaming in Germany, show that the average stay of a gambler in a town where gaming is allowed is ten days. This assertion has not so far been controverted by later statistics. It is obvious, therefore, that something more than games of chance are required to establish the popularity of a watering-place. The absence of gambling facilities in Germany has had no evil effect on the popularity of its health resorts, as is shown by the present condition of Wiesbaden, where 130,000 visitors are accommodated yearly. Thirty years ago the number was 40,000. This town is typical of many others in Germany, France, and Switzerland. The company which exploits the waters of Vichy-Etat sells twelve million bottles a year. In 1589, or 321 years ago, Spa, a Belgian watering-place, exported to the rest of Europe 300,000 pitchers of mineral waters, whereas to-day the sale of Spa-water is a negligible quantity. The lesson to be learnt, therefore, is that the prosperity of a health-resort depends in great measure on the enterprise of its authorities. If attractions are not provided and scientific exploitation neglected, its prosperity will decline despite its natural advantages.

### The Legal Responsibility of Opticians.

Into the long and bitterly controverted question of the fitness of the unqualified optician to prescribe glasses as well as to fit and make them we do not propose to enter again now; the matter has long ago been the subject of comment in these pages. A phase of the debated issue, however, has been recently prominent which is in many respects novel. We refer to the action brought by Miss Markham, a science student, against a Manchester optician for alleged negligence. The case was tried on October 24, 25, and 26; and the arguments are published *in extenso* in the *Times* and the *Ophthalmoscope*. Briefly what happened was as follows: The plaintiff was working very hard at the University of Wales for a degree in science at London University. In 1907 she found herself handicapped by visual symptoms, and went to the defendant, whose name is Thomas, trading as Wood-Abrahams. She was examined by an assistant, who ordered glasses after an examination which, according to Miss Markham, included the use of an ophthalmoscope; according to the defence a retinoscope only was used, but it was admitted that ophthalmoscopes are used in the shop. The distinction is not of any great importance. In 1908 and 1909 she returned, was again tested, and more glasses supplied. Eventually she consulted an ophthalmic surgeon, who discovered a fairly advanced conical cornea and an error of refraction three times as great as that corrected by the last glasses which the optician had supplied. On the ground that the conical cornea must have existed in 1907, and that by failing to detect it and to advise immediate cessation of study, the defendants had seriously prejudiced the plaintiff's sight and her chances of retaining such vision as she has, the action was brought for negligence. The judge, in summing up, pointed out to the jury that the defendant is not and has not pretended to be a professional ophthalmic surgeon; and that if people choose to go to an optician they do so at their own risk. An optician, however, he laid down, is bound to exercise reasonable care. In the end the jury disagreed and were discharged. With every sympathy for the plaintiff's misfortune, and with every desire to see curtailed the indiscriminate treatment of eye disorders by spectacle-makers, it is evident that there was no clear evidence of *mala praxis*. It is perhaps small consolation to Miss Markham to reflect that the example of her misfortune may save others from a similar fate; but from a strictly neutral standpoint it does seem as if that is the true moral of the case.

